# Anatomical and Technical Limitations of a Rabbit Median Sternotomy Model for Translational Mediastinal Adhesion Research

**DOI:** 10.3390/jcm15218616

**Published:** 2026-11-05

**Authors:** Doğuş Hemşinli, Sedat Ozan Karakişi, Atilla Topçu, Oğuzhan Okcu, İbrahim Yel, Fatih Eren

**Affiliations:** 1 Department of Cardiovascular Surgery, Faculty of Medicine, Recep Tayyip Erdoğan University, Rize 53100, Türkiye; dogus.hemsinli@erdogan.edu.tr (D.H.); ibrahim.yel@erdogan.edu.tr (İ.Y.); 2 Department of Pharmacology, Faculty of Medicine, Recep Tayyip Erdoğan University, Rize 53100, Türkiye; atilla.topcu@erdogan.edu.tr; 3 Department of Pathology, Faculty of Medicine, Recep Tayyip Erdoğan University, Rize 53100, Türkiye; oguzhan.okcu@erdogan.edu.tr; 4 Faculty of Medicine, Recep Tayyip Erdoğan University, Rize 53100, Türkiye; fatih.eren@erdogan.edu.tr; 5 Department of Medical Biology, Faculty of Medicine, Marmara University, Istanbul 34854, Türkiye; 6 Institute of Gastroenterology, Liver Research Unit, Marmara University, Istanbul 34854, Türkiye

**Keywords:** median sternotomy, mediastinal adhesions, rabbit model, surgical anatomy, translational research

## Abstract

**Background/Objectives:** Experimental animal models are fundamental to developing and translating strategies for preventing postoperative mediastinal adhesions. Rabbits are widely used in adhesion research; however, the anatomical and technical suitability of the rabbit median sternotomy model for reproducing conditions relevant to human redo sternotomy remains incompletely characterized. This study aimed to characterize the rabbit sternum and assess the feasibility and translational suitability of the rabbit median sternotomy model for mediastinal adhesion research. **Methods:** An acute, non-survival observational feasibility study was performed in two adult female New Zealand White rabbits. Median sternotomy was performed using 22 cm surgical scissors with ball-tipped blades, following a previously described technique with a technical modification. Macroscopic assessments included sternal morphology, the technical feasibility of midline division, pleural and peritoneal integrity, the apparent adequacy of the remaining sternal bone for re-approximation, and the relationships between the sternum and underlying mediastinal structures. Sternal closure was not attempted. **Results:** The rabbit sternum exhibited a narrow, highly segmented, and flexible configuration, with an elongated xiphoid process extending beyond the thoracic cavity. Median sternotomy proved technically challenging because of the limited width of the sternum. During the procedure, the surgical scissors deviated laterally, resulting in fragmentation of the sternal segments. Under the surgical conditions evaluated in this study, macroscopic assessment indicated that the remaining sternal bone was insufficient to permit stable re-approximation comparable to that required in humans; therefore, no closure attempt was made. **Conclusions:** These characteristics limited reproduction of the retrosternal conditions encountered during human median sternotomy. In this two-animal feasibility assessment, the rabbit median sternotomy model demonstrated anatomical and technical limitations that may reduce its translational fidelity for studies specifically intended to reproduce the retrosternal conditions of human redo sternotomy.

## 1. Introduction

Redo cardiac surgery is increasingly encountered in contemporary cardiac surgical practice as the population of patients with previous cardiac operations continues to grow [1,2]. Repeat cardiac surgery is associated with increased procedural complexity and perioperative risk, with intrathoracic adhesions representing a major contributor to prolonged dissection, bleeding, and potential injury to the heart and great vessels during re-entry [1,2,3,4,5,6,7,8]. In a series of 2555 patients undergoing repeat median sternotomy, injuries occurred in 9.0% of patients and were associated with substantially higher hospital mortality (18.5% vs. 6.5% in patients without injury); mortality reached 25% when injury occurred during sternal division [2]. The need for reoperation is also expected to remain clinically relevant as increasing numbers of patients undergo cardiac surgery at younger ages and as the use of bioprosthetic valves continues to expand, given their limited long-term durability [9,10,11]. The clinical significance of mediastinal adhesions in redo cardiac surgery is consequently increasing.

In recent years, considerable efforts have been directed toward the development of biomaterials, medical devices, pharmacological interventions, and surgical strategies aimed at preventing postoperative adhesions. The increasing clinical burden of redo cardiac surgery and the potentially severe consequences of resternotomy have consequently intensified the need for effective adhesion-prevention strategies and for experimental models that can reliably predict their clinical performance. Alternative surgical approaches have also been explored in an effort to avoid or reduce the hazards associated with repeat median sternotomy. However, establishing experimental models capable of reliably reproducing mediastinal adhesions while accurately reflecting the relevant human surgical anatomy remains a major challenge [12,13,14,15]. The existing literature reveals substantial heterogeneity in terms of animal species, surgical approaches, and outcome assessment methods. A systematic review of 41 studies on postoperative pericardial adhesions identified 42 animal models, including 18 studies involving 235 rabbits, and highlighted the heterogeneity of existing models as a barrier to standardization in this field [16].

Small-animal models are widely used in mediastinal adhesion research because of their practical advantages, and rabbits have been employed in several studies [4,13,15,16]. However, most rabbit models use thoracotomy to investigate intrapericardial adhesions, whereas studies specifically employing median sternotomy are relatively scarce [12,13,17,18,19]. Although the rabbit model has been recommended for mediastinal adhesion research [12], its anatomical and technical suitability for reproducing the conditions encountered during human redo sternotomy remains incompletely characterized. This distinction is important because anatomical differences between an experimental model and the clinical condition it is intended to represent may limit the translational interpretation of experimental findings.

The initial motivation for this study arose from the need to identify a suitable animal model for the potential future preclinical evaluation of a medical product intended to prevent postoperative mediastinal adhesions. The product is being developed through a separate TÜBİTAK-funded research project led by the corresponding author. A review of the literature identified rabbit median sternotomy as a potentially accessible and economical approach. Before proceeding to more extensive animal experiments, we therefore undertook a preliminary assessment of the anatomical and technical feasibility of this model. During this assessment, we identified limitations that raised concerns regarding its suitability for evaluating interventions intended to prevent retrosternal adhesions after cardiac surgery. Accordingly, the present study was confined to evaluating the animal model itself; the medical product was neither applied nor tested in this study.

Although rabbits have been used in experimental studies of mediastinal adhesion prevention, the anatomical and technical suitability of rabbit median sternotomy for reproducing conditions encountered during human redo sternotomy has not been adequately characterized. This is an important methodological consideration because the translational relevance of an experimental adhesion model depends not only on its suitability for surgical manipulation and adhesion assessment but also on the extent to which its anatomy represents the clinical setting under investigation. We therefore conducted a preliminary anatomical feasibility assessment to characterize rabbit sternal morphology, evaluate the technical feasibility of median sternotomy using a previously described approach with a technical modification, and determine whether the resulting surgical anatomy adequately represents conditions relevant to human redo sternotomy. By identifying potential anatomical and technical limitations before undertaking more extensive experiments, this study aimed to inform the selection and validation of appropriate animal models for translational mediastinal adhesion research.

## 2. Materials and Methods

### 2.1. Study Design

This study was conducted following approval by the Animal Experiments Local Ethics Committee (HADYEK) of Recep Tayyip Erdoğan University (Approval No. 2025/23, dated 8 May 2025). It was designed as an observational feasibility study to evaluate the anatomical and technical feasibility of median sternotomy in the rabbit model, rather than as a comparative hypothesis-testing experiment. Previous studies have employed rabbit models of postoperative pericardial or retrosternal adhesion formation using median sternotomy, with some procedures performed under spontaneous respiration without endotracheal intubation or mechanical ventilation [12,13,17,18,19]. However, these studies primarily investigated adhesion formation and the effectiveness of preventive interventions rather than the anatomical and technical feasibility of the sternotomy procedure itself. The present study specifically examined whether the scissors-based approach could adequately reproduce the anatomical conditions relevant to human median sternotomy. The two-animal sample was deliberately selected to identify fundamental anatomical and technical limitations before undertaking more extensive animal experiments, in accordance with the principle of Reduction. The objective was not to establish the prevalence of these findings across the rabbit population, but to assess the feasibility of the specific surgical approach under the conditions evaluated.

The primary objectives were to characterize the anatomical features encountered during median sternotomy in rabbits, assess the technical feasibility of the procedure, and macroscopically evaluate the suitability of the rabbit sternum for use in median sternotomy-based models of postoperative mediastinal adhesions. No prospective protocol registration was performed. The study methods and procedures were described in the research proposal submitted to the institutional ethics committee and the university’s BAP unit before the study commenced.

### 2.2. Experimental Animals

All experimental procedures were conducted in accordance with the ARRIVE 2.0 guidelines [20]. The animals were obtained through the Animal Experiments Local Ethics Committee (HADYEK) of Recep Tayyip Erdoğan University. Both rabbits were purpose-bred, healthy adult female New Zealand White rabbits, approximately one year old and weighing 4.5–4.8 kg. Their health status and suitability for experimental procedures were documented in the relevant veterinary and institutional records. Neither animal had undergone previous surgical procedures. All animal procedures were performed in compliance with national and international guidelines for the care and use of laboratory animals. The rabbits were housed in standard cages under controlled environmental conditions, with an ambient temperature of 20–24 °C and relative humidity of 55–65%, and in a 12-h light/dark cycle. Both animals were provided with ad libitum access to standard laboratory chow and tap water before the study.

### 2.3. Surgical Procedure

Both rabbits received intramuscular ketamine hydrochloride (Ketalar; Pfizer İlaçları Ltd. Şti., İstanbul, Türkiye) at a dose of 35 mg/kg and xylazine hydrochloride (Rompun; Bayer Türk Kimya San. Ltd. Şti., İstanbul, Türkiye) at a dose of 5 mg/kg. Endotracheal intubation and mechanical ventilation were not performed, and both animals breathed spontaneously throughout the procedures. Following confirmation of an adequate depth of anesthesia, the animals were placed in the supine position. The operative field was then prepared and draped under standard sterile conditions. Anaesthetic depth was assessed every 5 min using the pedal withdrawal reflex, and additional anaesthetic was administered when necessary. No observable responses indicating inadequate anaesthesia or pain-related reactions were noted. No additional analgesic medication or intravenous fluid support was provided. Body temperature was maintained using a heating lamp. As this was an acute, non-survival feasibility study, no postoperative recovery period was planned.

**Figure 1:**
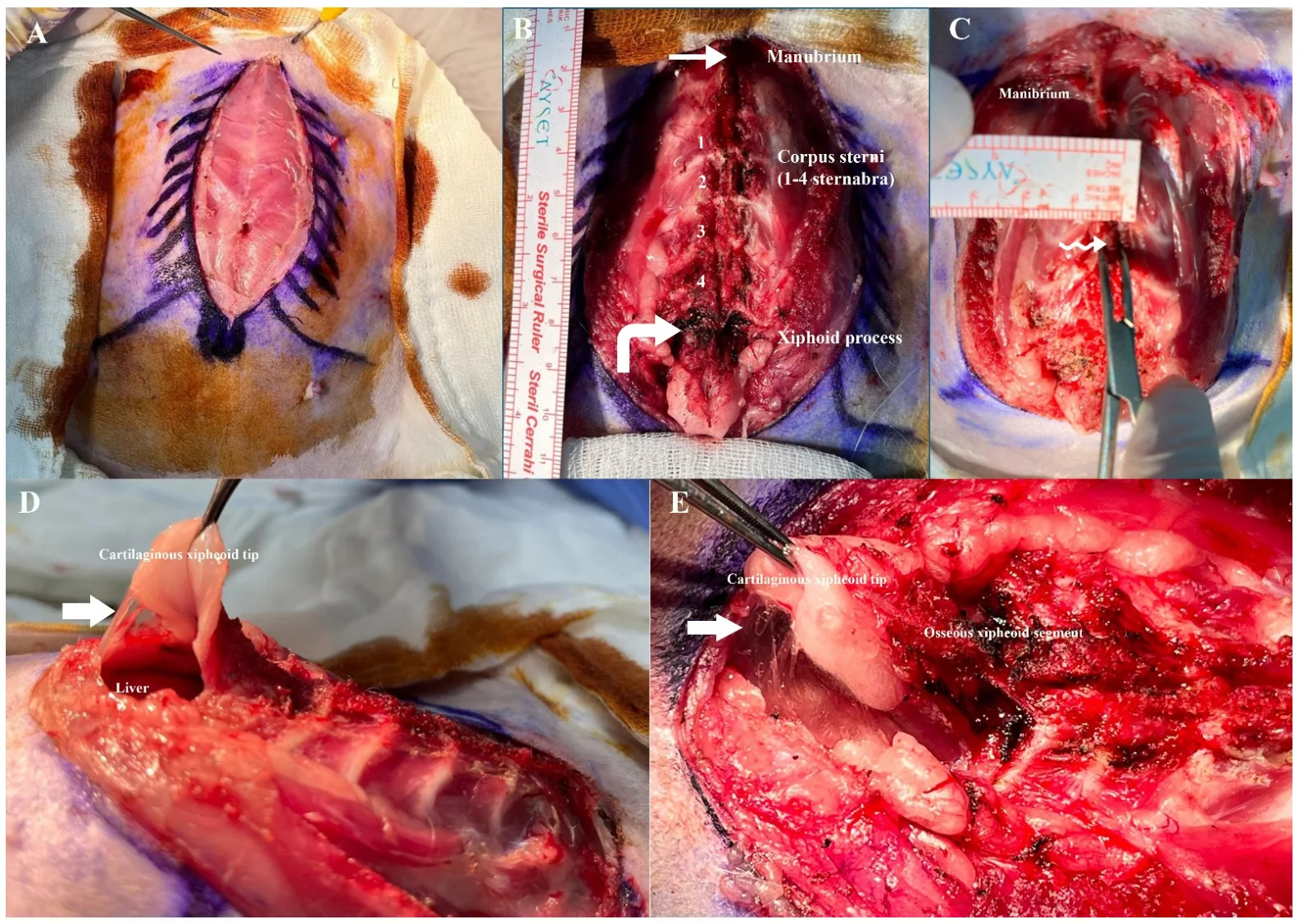
(**A**) Midline skin incision and subcutaneous dissection exposing the sternum. (**B**) Exposed rabbit sternum showing the manubrium, four sternebrae, and xiphoid process. The thin arrow indicates the cranial manubrium and the curved arrow the xiphoid process. (**C**) Magnified sternum. The double wavy arrow indicates the narrow corpus sterni, with a transverse width and anteroposterior thickness of approximately 3 mm. (**D**) Caudal view of the xiphoid process. The thick arrow indicates the thin parietal peritoneum. (**E**) Magnified xiphoid process. The thick arrow indicates the parietal peritoneum.

A midline skin incision was made from the cranial aspect of the sternum to the distal tip of the xiphoid process (Figure 1A). The subcutaneous tissues were carefully dissected using electrocautery (ES350 electrosurgical unit; EMED Sp. z o.o. sp. k., Opacz-Kolonia, Poland). (Figure 1B,C). The anatomical segments of the sternum, their morphological characteristics, and their relationships with adjacent anatomical structures were systematically examined and documented using high-resolution digital photography (Figure 1B–E).

**Figure 2:**
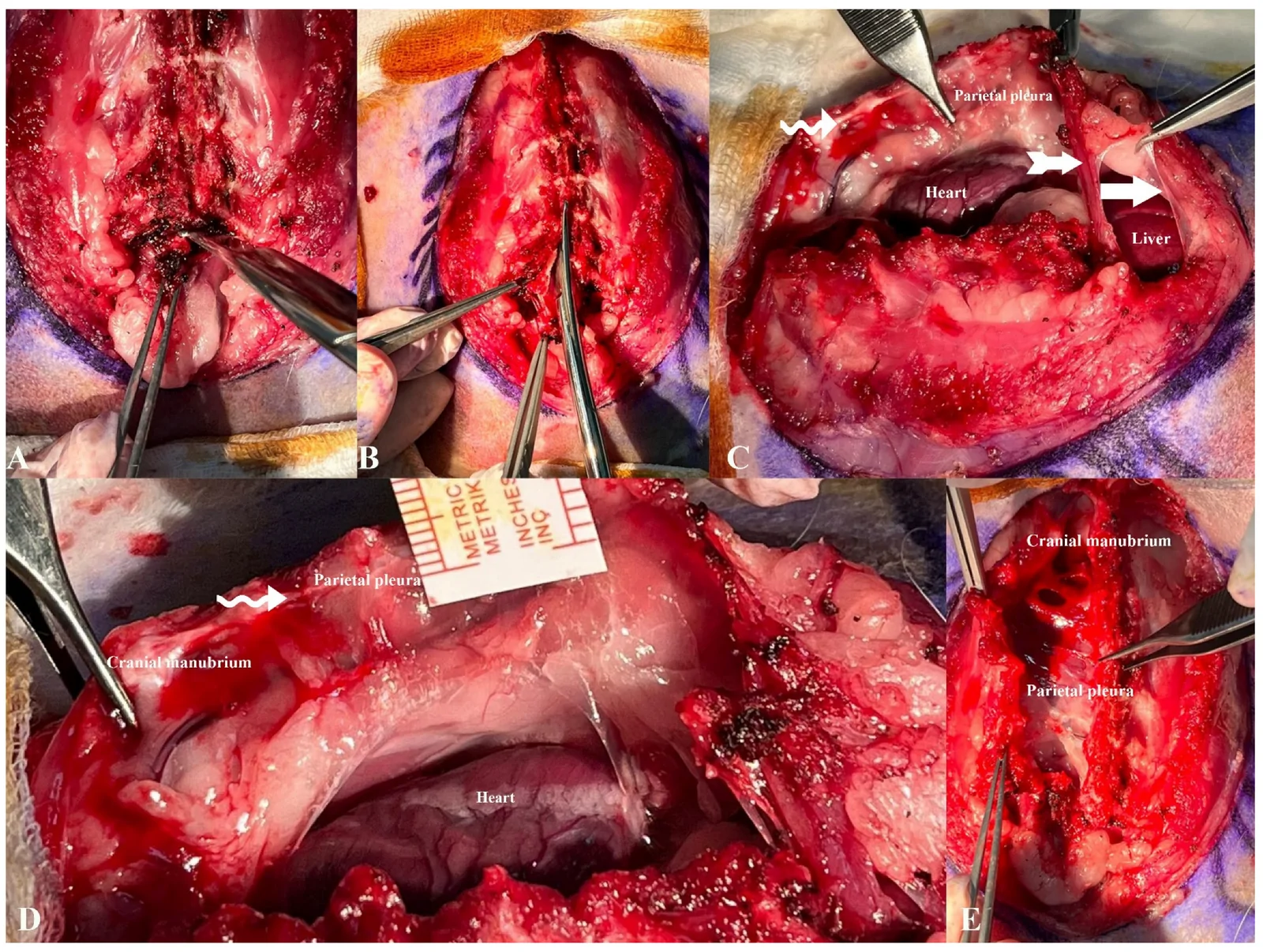
(**A**) Posterior entry at the corpus sterni–xiphoid junction before median sternotomy. (**B**) Median sternotomy performed from caudal to cranial using surgical scissors. (**C**) Thoracic cavity after median sternotomy. The notched arrow indicates the diaphragm, the thick arrow the parietal peritoneum and the double wavy arrow the mediastinal surface of the sternum. (**D**) Magnified mediastinal surface. The double wavy arrow indicates the mediastinal surface of the sternum. (**E**) Superior (surgeon’s) view after median sternotomy.

**Figure 3:**
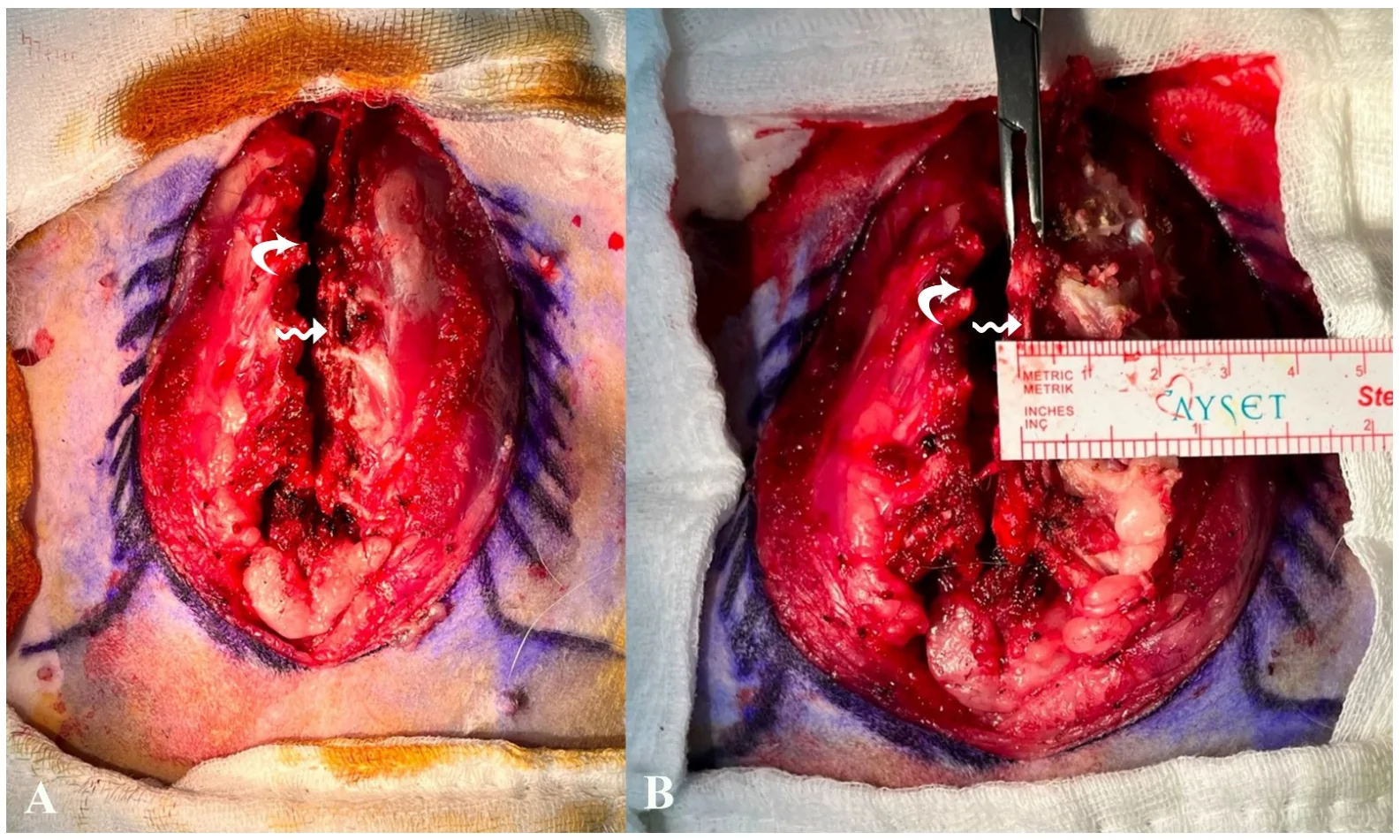
(**A**) Superior (surgeon’s) view after median sternotomy. The double wavy arrow indicates compressed and disrupted sternebrae, and the curved arrow indicates disrupted sternocostal junctions with exposed rib ends. (**B**) Close-up of the sternum after attempted midline division. The double wavy arrow indicates compressed and fragmented sternebrae, and the curved arrow indicates disrupted sternocostal junctions.

A median sternotomy was performed in both rabbits using standard 22-cm surgical scissors with ball-tipped blades (Bahadır Surgical Instruments, Samsun, Türkiye). The xiphoid process was managed differently in the two animals. In the first rabbit, the sternotomy included the xiphoid region. In the second rabbit, following the approach described by Okuyama et al. [12], the xiphoid process was detached from the sternal body before the sternotomy (Figure 2A). The scissors were then advanced cranially along the sternal body (corpus sterni). Unlike the original technique, in which the sternotomy terminates at the level of the second rib and the incision is maintained open using a small retractor, the sternotomy in the present study was extended cranially through the manubrium to expose the entire sternum and permit comprehensive anatomical assessment (Figure 2B). During the procedure, the feasibility of controlled midline separation, the integrity of the sternal bone, pleural and peritoneal integrity, and the suitability of the remaining sternal bone for re-approximation were assessed macroscopically by the cardiac surgical team, and a consensus was reached regarding their interpretation. The findings were documented using high-resolution digital photographs (Figure 2C–E and Figure 3A,B). Sternal closure was not attempted because the study was designed as an acute, non-survival anatomical feasibility assessment.

Following sternotomy, a longitudinal pericardiotomy was performed to permit macroscopic examination of the anatomical relationships between the sternum, heart, ascending aorta, and other mediastinal structures. The pericardium was not closed because the study was designed as an acute, non-survival anatomical feasibility assessment, and no postoperative recovery or survival period was planned. These anatomical relationships were documented using high-resolution digital photography (Figure 2C–E). At the end of each procedure, both animals were euthanized under deep anesthesia by exsanguination following right-atrial blood collection. Anesthetic depth was monitored throughout the procedure by assessing the pedal withdrawal reflex at 5-min intervals, with additional anesthetic administered when necessary. Before exsanguination, anesthetic depth was confirmed by the absence of a pedal withdrawal response. Blood collection was performed solely as part of the euthanasia procedure and not for a separate analytical investigation. Given the descriptive anatomical feasibility design, the absence of experimental groups, and the lack of comparative statistical analyses, blinding was not applicable to group allocation or data analysis. The surgical procedures were performed by two investigators, and the anatomical and technical findings were jointly assessed by the cardiac surgeons involved in the study, with consensus reached regarding their interpretation. Blinding was not applicable to this descriptive feasibility study. Following the experimental procedures, the sternums were preserved in formaldehyde for subsequent anatomical examination.

## 3. Results

### 3.1. Macroscopic Anatomical Findings

**Figure 4:**
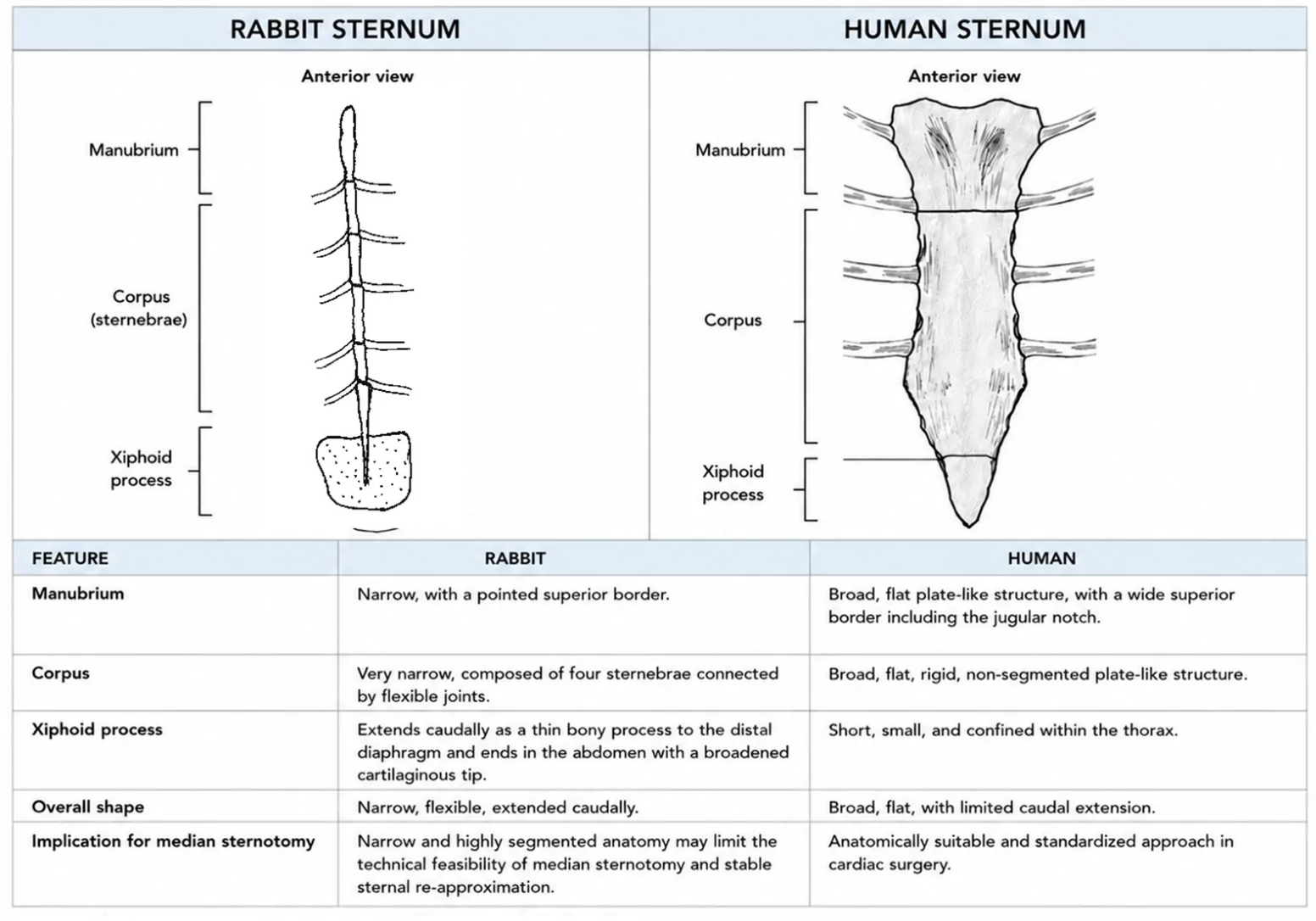
Comparative schematic representation of rabbit and human sternal anatomy, highlighting structural features relevant to sternotomy. Original illustrations created by the authors; anatomical features were verified against the anatomical descriptions provided in Refs. [21,22]. The dotted area represents the cartilaginous portion.

Macroscopic examination revealed that the rabbit sternum was narrow, flexible, and highly segmented, differing substantially in morphology from the broad, relatively rigid human sternum (Figure 4) [21,22]. The manubrium and individual sternebrae measured approximately 3 mm in both transverse width and anteroposterior thickness (Figure 1C, Figure 2B–E and Figure 3A,B). The manubrium sterni exhibited a narrow morphology that tapered progressively toward a pointed cranial end, whereas the human manubrium is broad and flat (Figure 1C and Figure 2E). The corpus sterni comprised four sternebrae connected sequentially by intersternebral joints (Figure 1B). The ribs articulated with the sternum laterally at the sternocostal junctions. Caudal to the corpus sterni, the xiphoid process continued as a slender bony segment extending beyond the diaphragm and terminating in a broad cartilaginous plate in the upper abdominal region (Figure 1B–E). Individual measurements for both animals are presented in Table 1.

The xiphoid plate was closely apposed to the extremely thin, translucent peritoneum (Figure 1D,E and Figure 2C). The right and left pleural reflections were separated by approximately 2 mm at the midline (Figure 2C–E). These anatomical relationships resulted in limited separation of the peritoneal and pleural structures from the operative field during median sternotomy. Although several measurements were identical in the two rabbits, some differences were observed in individual sternal segment lengths. These animal-specific measurements are presented separately in Table 1. Peritoneal integrity differed between the two animals. In the first rabbit, inclusion of the xiphoid region in the sternotomy resulted in peritoneal injury. In the second rabbit, the peritoneum remained intact after the xiphoid component had been separated from the sternal body before sternotomy.

### 3.2. Technical Findings During Median Sternotomy

Due to its markedly narrower configuration, the rabbit sternum did not permit a scissors-based division that adequately reproduced human median sternotomy. When the scissors deviated laterally, they moved toward the sternocostal junctions; in both rabbits, leftward deviation disrupted these joints. When the scissors remained near the midline, compression and fragmentation of the bony sternebrae occurred rather than separation of the intersternebral joints (Figure 2B,E and Figure 3A,B). Both rabbits developed left-sided pneumothoraxes, highlighting the technical difficulty of achieving a controlled midline division while preserving sternal and thoracic integrity. Additionally, progressive narrowing of the manubrium toward its cranial end prevented initiation of the sternotomy cranially, as is standard in human surgery (Figure 1B,C and Figure 2D,E).

### 3.3. Sternal Re-Approximation and Mediastinal Relationships

Following sternotomy, macroscopic assessment indicated that the remaining sternal bone was insufficient in both width and structural integrity to permit stable re-approximation (Figure 2C–E and Figure 3A,B). As this study was designed as an acute, non-survival assessment of anatomical feasibility, sternal closure was not attempted. Consequently, the feasibility of chest-wall closure using pericostal or absorbable sutures could not be determined.

Following pericardiotomy, the heart, ascending aorta, and other mediastinal structures were visualized, and their anatomical relationships to the sternum were assessed (Figure 2C,D). The observed anatomy was characterized by a narrow, highly segmented sternum, close proximity between the sternum and adjacent thoracic and upper abdominal structures, and residual sternal bone that was considered insufficient for stable re-approximation using techniques comparable to those employed for human sternal closure.

## 4. Discussion

Mediastinal adhesions remain a major challenge in redo cardiac surgery, increasing the risk of cardiac and great vessel injury during resternotomy and retrosternal dissection, as well as bleeding, transfusion requirements, intensive care unit and hospital stays, and healthcare costs [23,24,25,26]. Despite decades of research, no strategy for preventing postoperative mediastinal adhesions has achieved widespread acceptance in routine clinical practice [5,16]. Consequently, the development of effective preventive interventions requires experimental models that adequately reproduce the anatomical conditions encountered during human redo sternotomy.

The principal finding of this two-animal feasibility assessment is that anatomical and technical features of rabbit median sternotomy may limit its translational fidelity for reproducing human redo sternotomy (Figure 4). Rather than investigating adhesion formation, this study addressed a more fundamental methodological question: whether the rabbit sternum and the resulting surgical environment adequately represent the anatomical conditions relevant to human resternotomy. Our findings support critical anatomical validation of this model before its use in translational studies of retrosternal adhesion prevention.

The rabbit sternum, comprising a manubrium, four sternebrae forming the corpus sterni, and an elongated xiphoid process, differs from the human sternum in its narrow, segmented, and relatively flexible structure. The xiphoid process extends beyond the diaphragm and lies close to the peritoneum, while the pleural cavities are immediately adjacent along the midline. Together with the limited width of the midline bony surface, these features make controlled median sternotomy technically challenging in rabbits [21]. Although these anatomical characteristics may influence surgical access and chest-wall reconstruction, their effects on postoperative healing and mediastinal adhesion formation were not assessed in the present study.

Stable sternal closure is important for chest-wall stability and osseous healing. In humans, the sternum has mean widths of 38.2–39.8 mm at the manubrium and 26.7–28.3 mm at the sternal body, with mean thicknesses of 8.5–12.1 mm depending on anatomical region and sex [27]. Despite these dimensions, sternal dehiscence remains a clinically significant complication after cardiac surgery. In the present study, the narrow, segmented, and relatively flexible rabbit sternum made controlled midline division difficult and left insufficient bone for macroscopic approximation comparable to conventional human sternal closure. As no closure attempt was performed, the feasibility of alternative chest-wall closure techniques could not be assessed, and the implications for postoperative healing remain unknown. These findings reflect the specific rabbit anatomy and scissors-based approach evaluated and should not be interpreted as evidence that closure is infeasible with all techniques. Accordingly, the suitability of this model for studying human retrosternal adhesions should be distinguished from its potential utility in broader investigations of adhesion formation.

In a review of 41 experimental studies of postoperative pericardial adhesions, rabbits were the most frequently used model, accounting for 44% of studies (18/41) and 57% of the animals included (235/412) [16]. Their popularity has been attributed to practical advantages, including their relatively large size among small-animal models, ease of surgical manipulation, low cost, and reportedly low perioperative mortality [12,13,17,18,19]. However, studies specifically employing median sternotomy are comparatively limited. Among the studies identified, Okuyama et al. provided a detailed description of the surgical technique [12], whereas the present study evaluated a modified version of their approach. Therefore, the technical difficulties observed here cannot establish whether the original technique would produce different results. More broadly, the widespread use of rabbits in adhesion research does not, by itself, establish their anatomical suitability for reproducing human redo sternotomy.

The narrow sternal anatomy presented two principal technical challenges. Lateral deviation of the scissors produced an asymmetric opening, with sternal bone remaining on one side and rib ends and intercostal spaces forming much of the opposing margin. Conversely, maintaining the scissors near the midline resulted in crushing and fracture of the thin sternal bone. Neither outcome reproduced the clean, symmetrical division achieved with a dedicated sternotomy saw in human cardiac surgery. These findings demonstrate limitations of the scissors-based approach evaluated but do not establish that median sternotomy is universally infeasible in rabbits.

Previous studies have reported variable levels of methodological detail concerning sternotomy, preservation of pleural and peritoneal integrity, sternal closure, and postoperative healing. Detailed intraoperative images allowing independent evaluation of the anatomical feasibility of the described procedures have also been limited [12,13,17,18,19]. Notably, Çolak et al. and Hu et al. described sternotomy extending from the xiphoid process to the jugular notch. However, rabbits do not have a distinct jugular notch comparable to that of humans, making the anatomical landmarks used to describe this approach difficult to interpret in direct human anatomical terms [13,17]. Ohno et al. used a circular saw and closed the sternum with 2-0 braided silk sutures [28], while Kasimzade et al. and Amal et al. reported postoperative sternal healing using their respective approaches [29,30]. These reports indicate that sternal closure and healing have been achieved under certain experimental conditions, but differences in instruments, techniques, closure methods, and animal characteristics limit direct comparisons.

Alternative approaches, including an appropriately scaled oscillating micro-saw or division through the intersternebral cartilage, might improve the precision of sternal division or reduce bone trauma. However, neither approach was evaluated in the present study, and their suitability for preserving sufficient structural integrity for stable re-approximation remains unknown. Likewise, the reported success of closure and healing in other studies does not establish whether the resulting postoperative anatomy reproduces the anatomical and biomechanical conditions relevant to human redo sternotomy. The translational relevance of rabbit median sternotomy therefore requires evaluation beyond the technical feasibility of performing the incision itself.

These findings have implications for the selection of experimental models used to evaluate novel biomaterials, medical devices, and surgical approaches for preventing postoperative mediastinal adhesions. The rabbit remains a potentially valuable model for thoracotomy-based adhesion studies and investigations of the inflammatory mechanisms underlying adhesion formation. However, when the research objective is to reproduce the anatomical conditions encountered during human redo sternotomy, its median sternotomy model should not be assumed to be anatomically interchangeable with the human setting without appropriate validation.

Early anatomical assessment is also relevant to the ethical conduct of animal research. In accordance with the 3R principles (Replacement, Reduction, and Refinement), identifying substantial limitations before proceeding to larger experiments may help prevent unnecessary animal use and inform more appropriate study design. The present study illustrates the importance of assessing the anatomical and technical suitability of an experimental model before proceeding to more extensive preclinical investigations of interventions intended to prevent postoperative mediastinal adhesions.

Taken together, these findings highlight an important methodological consideration in mediastinal adhesion research: the extensive use of an animal model does not establish its anatomical suitability for the specific clinical condition being investigated. Anatomical fidelity should therefore be considered alongside practical advantages when selecting models for translational evaluation of interventions intended to prevent adhesions associated with human redo sternotomy.

## 5. Limitations

This study has several limitations. First, only two approximately one-year-old female New Zealand White rabbits weighing 4.5–4.8 kg were examined. The small sample size was intentional because this was a preliminary anatomical feasibility assessment designed to identify potential model limitations, rather than a hypothesis-testing experiment. In accordance with the 3R principles, animal use was minimized; however, the findings remain descriptive and cannot establish inter-animal variability or be generalized confidently to males, other rabbit strains, or animals of different ages and sizes.

Second, the acute, non-survival design permitted only intraoperative macroscopic assessment. Postoperative sternal healing, bone remodeling, long-term retrosternal reconstruction, and mediastinal adhesion formation were not evaluated. In addition, no quantitative biomechanical testing, advanced imaging, or histological examination was performed.

Third, all procedures were performed by the same research team using 22-cm straight surgical scissors with ball-tipped blades and a technically modified version of the approach described by Okuyama et al. [12]. The surgeon had no prior experience with rabbit sternotomy. Consequently, the individual contributions of operator experience, instrument selection, and surgical technique to the observed difficulties cannot be distinguished. The findings therefore apply to the specific approach evaluated and do not establish that median sternotomy is universally infeasible in rabbits.

Finally, sternal closure and alternative closure techniques were not attempted, and no direct comparison with other animal models, including large-animal models, was performed. The study therefore cannot determine whether alternative instruments or closure methods could overcome the observed limitations, or whether another model would better reproduce the human redo sternotomy environment. Further studies incorporating alternative surgical approaches, objective anatomical and biomechanical assessments, and postoperative follow-up are needed to address these questions.

## 6. Conclusions

Based on our direct anatomical observations, rabbit median sternotomy presents anatomical and technical limitations that may reduce its ability to reproduce the conditions encountered during human redo sternotomy. Although rabbits have been used in retrosternal adhesion research, and previous studies have highlighted practical advantages such as spontaneous breathing without mechanical ventilation and relatively low postoperative morbidity and mortality, these benefits should not be assumed to apply uniformly across surgical settings. In our two-animal feasibility assessment, the narrow, segmented sternum and difficulties with midline division raised concerns regarding the suitability of this model for studies requiring anatomically representative median sternotomy.

These findings do not imply that rabbits are inherently unsuitable for adhesion research; rather, model selection should reflect the specific anatomical and clinical scenario under investigation. The reported advantages of rabbit sternotomy should be balanced against procedure-specific anatomical and technical challenges, particularly when considering the 3R principles. Comprehensive anatomical validation and transparent reporting of surgical feasibility, complications, and closure outcomes are therefore warranted before this model is adopted for translational studies of mediastinal adhesion prevention. The implications of the observed findings for postoperative healing and adhesion formation remain hypothetical and require appropriately designed survival studies.

## Figures and Tables

**Table 1:** Individual sternal and mediastinal morphometric measurements in the two rabbits.

Parameter	Rabbit 1	Rabbit 2	Unit
Body weight	4.5	4.8	kg
Total sternal length	96	99	mm
Manubrium sterni—length	20	21	mm
Manubrium sterni—width	3	3	mm
Manubrium sterni—anteroposterior thickness	3	3	mm
First sternebra—length	13	13	mm
First sternebra—width	3	3	mm
First sternebra—anteroposterior thickness	3	3	mm
Second sternebra—length	13	13	mm
Second sternebra—width	3	3	mm
Second sternebra—anteroposterior thickness	3	3	mm
Third sternebra—length	12	13	mm
Third sternebra—width	3	3	mm
Third sternebra—anteroposterior thickness	3	3	mm
Fourth sternebra—length	8	9	mm
Fourth sternebra—width	3	3	mm
Fourth sternebra—anteroposterior thickness	3	3	mm
Total xiphoid length	30	30	mm
Osseous xiphoid—length	17	18	mm
Osseous xiphoid—anteroposterior thickness	2	2	mm
Cartilaginous terminal plate—length	13	12	mm
Cartilaginous terminal plate—width	25	26	mm
Cartilaginous terminal plate—anteroposterior thickness	2	2	mm
Midline distance between the pleural reflections	2	2	mm

## Data Availability

The data supporting the findings of this study are available from the corresponding author upon reasonable request. The complete photographic documentation of the anatomical observations and technical findings from both animals is provided in the Supplementary Materials.

## Supplementary Materials

The following supporting information can be downloaded at: https://www.mdpi.com/article/10.3390/jcm15218616/s1. The complete photographic documentation of the anatomical observations and technical findings from both rabbits is provided in the Supplementary Materials as Supplementary Figures.

## References

[1] Minns S., Tosh W., Moorjani N. (2024). Anaesthesia for Adult Cardiac Surgery Requiring Repeat Sternotomy. BJA Educ..

[2] Park C.B., Suri R.M., Burkhart H.M., Greason K.L., Dearani J.A., Schaff H.V., Sundt T.M. (2010). Identifying Patients at Particular Risk of Injury during Repeat Sternotomy: Analysis of 2555 Cardiac Reoperations. J. Thorac. Cardiovasc. Surg..

[3] Lu J.H., Chang Y., Sung H.W., Chiu Y.T., Yang P.C., Hwang B. (1998). Heparinization on Pericardial Substitutes Can Reduce Adhesion and Epicardial Inflammation in the Dog. J. Thorac. Cardiovasc. Surg..

[4] Saha K., McEwan W., Frizelle F.A., Singh H. (2004). Limiting Pericardial Adhesions after Coronary Artery Bypass: Experimental Study. Asian Cardiovasc. Thorac. Ann..

[5] Alizzi A.M., Summers P., Boon V.H., Tantiongco J.P., Thompson T., Leslie B.J., Williams D., Steele M., Bidstrup B.P., Diqer A.M.A. (2012). Reduction of Post-Surgical Pericardial Adhesions Using a Pig Model. Heart Lung Circ..

[6] Biçer M., Bayram A.S., Gürbüz O., Şenkaya I., Yerci Ö., Tok M., Anǧ E., Moǧol E.B., Saba D. (2008). Assesssment of the Efficacy of the Bio-Absorbable Oxidized Regenerated Cellulose for Prevention of Post-Operative Pericardial Adhesion in the Rabbit Model. J. Int. Med. Res..

[7] Chen Z., Zheng J., Zhang J., Li S. (2015). A Novel Bioabsorbable Pericardial Membrane Substitute to Reduce Postoperative Pericardial Adhesions in a Rabbit Model. Interact. Cardiovasc. Thorac. Surg..

[8] Connors R.C., Muir J.J., Liu Y., Reiss G.R., Kouretas P.C., Whitten M.G., Sorenson T.K., Prestwich G.D., Bull D.A. (2007). Postoperative Pericardial Adhesion Prevention Using Carbylan-SX in a Rabbit Model. J. Surg. Res..

[9] Cesnjevar R., Purbojo A., Haake C., Laas J. (2022). Significant Adhesion Reduction and Time Saving in Pediatric Heart Surgery with 4DryField PH: A Retrospective, Controlled Study. PLoS ONE.

[10] Ghoreishi M., Dawood M., Hobbs G., Pasrija C., Riley P., Petrose L., Griffith B.P., Gammie J.S. (2013). Repeat Sternotomy: No Longer a Risk Factor in Mitral Valve Surgical Procedures. Ann. Thorac. Surg..

[11] Di Mauro M., Iacò A.L., Contini M., Teodori G., Vitolla G., Pano M., Di Giammarco G., Calafiore A.M. (2005). Reoperative Coronary Artery Bypass Grafting: Analysis of Early and Late Outcomes. Ann. Thorac. Surg..

[12] Okuyama N., Rodgers K.E., Wang C.Y., Girgis W., Oz M., St. Amand K., Pines E., Decherney A.H., Rose E.A., Cohn D. (1998). Prevention of Retrosternal Adhesion Formation in a Rabbit Model Using Bioresorbable Films of Polyethylene Glycol and Polylactic Acid. J. Surg. Res..

[13] Hu C., Tang F., Wu Q., Guo B., Long W.A., Ruan Y., Li L. (2021). Novel Trilaminar Polymeric Antiadhesion Membrane Prevents Postoperative Pericardial Adhesion. Ann. Thorac. Surg..

[14] Kojima A., Sakaue T., Okazaki M., Shikata F., Kurata M., Imai Y., Nakaoka H., Masumoto J., Uchita S., Izutani H. (2019). A Simple Mouse Model of Pericardial Adhesions. J. Cardiothorac. Surg..

[15] Kang H., Chung Y.S., Kim S.W., Choi G.J., Kim B.G., Park S.W., Seok J.W., Hong J. (2015). Effect of Temperature-Sensitive Poloxamer Solution/Gel Material on Pericardial Adhesion Prevention: Supine Rabbit Model Study Mimicking Cardiac Surgery. PLoS ONE.

[16] Hill M.A., Walkowiak O.A., Head W.T., Kwon J.H., Kavarana M.N., Rajab T.K. (2022). A Review of Animal Models for Post-Operative Pericardial Adhesions. Front. Surg..

[17] Colak N., Nazli Y., Alpay M.F., Aksoy O.N., Akkaya I.O., Bayrak R., Cakir O. (2013). Effect of Topical N-Acetylcysteine in the Prevention of Postoperative Pericardial Adhesion Formation in a Rabbit Model. Cardiovasc. Pathol..

[18] Krause T.J., Zazanis G., Malatesta P., Solina A. (2001). Prevention of Pericardial Adhesions with N-O Carboxymethylchitosan in the Rabbit Model. J. Investig. Surg..

[19] Zhou J., Liwski R.S., Elson C., Lee T.D.G. (2008). Reduction in Postsurgical Adhesion Formation after Cardiac Surgery in a Rabbit Model Using N,O-Carboxymethyl Chitosan to Block Cell Adherence. J. Thorac. Cardiovasc. Surg..

[20] Percie du Sert N., Hurst V., Ahluwalia A., Alam S., Avey M.T., Baker M., Browne W.J., Clark A., Cuthill I.C., Dirnagl U. (2020). The ARRIVE Guidelines 2.0: Updated Guidelines for Reporting Animal Research. Br. J. Pharmacol..

[21] Chin E. (1957). The Rabbit: An Illustrated Anatomical Guide. Master’s Thesis.

[22] Agur A.M.R., Dalley A.F. (2021). Grant’s Atlas of Anatomy.

[23] Elbadawi A., Hamed M., Elgendy I.Y., Omer M.A., Ogunbayo G.O., Megaly M., Denktas A., Ghanta R., Jimenez E., Brilakis E. (2020). Outcomes of Reoperative Coronary Artery Bypass Graft Surgery in the United States. J. Am. Heart Assoc..

[24] Gallo M., Trivedi J.R., Monreal G., Ganzel B.L., Slaughter M.S. (2020). Risk Factors and Outcomes in Redo Coronary Artery Bypass Grafting. Heart Lung Circ..

[25] Bhamidipati C.M., LaPar D.J., Fonner E., Kern J.A., Kron I.L., Ailawadi G. (2011). Outcomes and cost of cardiac surgery in octogenarians is related to type of operation: A multi-institutional analysis. Ann. Thorac. Surg..

[26] Lefort B., El Arid J.M., Bouquiaux A.L., Soulé N., Chantreuil J., Tavernier E., Chantepie A., Neville P. (2015). Is Seprafilm Valuable in Infant Cardiac Redo Procedures?. J. Cardiothorac. Surg..

[27] Kirum G.G., Kukiriza J., Tumusiime G. (2020). A Morphometric Study of Adult Human Sterna from the Galloway Osteological Collection. Acad. Anat. Int..

[28] Ohno T., Suenaga H., Yamawaki-Ogata A., Kanie K., Kato R., Uto K., Ebara M., Ito H., Narita Y., Usui A. (2023). Development of Novel Waxy Bone Haemostatic Agents Composed of Biodegradable Polymers with Osteogenic-Enhancing Peptides in Rabbit Models. Interdiscip. Cardiovasc. Thorac. Surg..

[29] Kasimzade F., Ada F., Karatas O. (2022). Evaluation of a Sternum Dehiscence Reconstruction Graft on an Animal Model. Med. Sci. Int. Med. J..

[30] Amal I., Soebroto H. (2021). Puruhito Comparison of Bone Wax and Chitosan Usage on Post-Sternotomy Bone Healing. Asian Cardiovasc. Thorac. Ann..

